# Expression and Prognostic Role of PANK1 in Glioma

**DOI:** 10.2174/1386207326666230502103726

**Published:** 2024-03-07

**Authors:** Zhiming Zhao, Xu Xu, Shijing Ma, Li Li

**Affiliations:** 1 Department of Geriatrics, Renmin Hospital of Wuhan University, Wuchang District, Wuhan, 430060, China

**Keywords:** PANK1, glioma, biomarker, prognosis, invasion *in vitro* assays, glioblastoma multiform

## Abstract

**Background:**

Malignant gliomas are the most common type of primary malignant brain tumors. Pantothenate kinase 1 (PANK1) mRNA is highly expressed in several metabolic processes, implying that PANK1 plays a potential role in metabolic programming in cancers. However, the role of PANK1 in glioma has not been fully explored.

**Methods:**

Public datasets (The Cancer Genome Atlas (TCGA), Chinese Glioma Genome Atlas (CGGA), Gravendeel and Rembrandt) and validation cohort were used to explore the expression of PANK1 in glioma tissues. Kaplan–Meier and Cox regression analyses were used to explore the relationship between PANK1 and prognosis in glioma. Cell proliferation and invasion were determined using Cell Counting Kit-8 (CCK8) and transwell invasion *in vitro* assays.

**Results:**

Analysis using the four public datasets and the validation cohort showed that PANK1 expression was significantly downregulated in glioma tissues compared with non-tumor tissues (*P<*0.01). PANK1 expression was negatively correlated with World Health Organization (WHO) grade, 1p/19q non-codeletion and isocitric dehydrogenase 1/2 (IDH1/2) wildtype. Furthermore, high expression of PANK1 was correlated with significantly better prognosis of glioma patients compared to patients with low expression of PANK1 (all *P<*0.01 in the four datasets). Besides, both lower-grade glioma (LGG) and glioblastoma multiform (GBM) patients with high expression of PANK1 had a significantly better prognosis than those with low expression of PANK1 in TCGA, Gravendeel and Rembrandt datasets (all *P <*0.01). Multivariate Cox regression analysis revealed that low PANK1 expression was an independent risk factor associated with a worse prognosis of glioma patients. Moreover, overexpression of PANK1 significantly inhibited the proliferation and invasion of U87 and U251 cells.

**Conclusion:**

PANK1 expression is downregulated in glioma tissues and is a novel prognostic biomarker in glioma patients.

## 1 INTRODUCTION

Glioma is the most common tumor of the central nervous system. Glioma accounts for 80% of all malignant brain tumors [1]. Previous studies reported that the median survival time of patients with grade IV glioblastoma multiform (GBM) is only 1 year, and the 5-year survival rate is less than 5%; thus, glioma is one of the malignant tumors with the highest mortality [2]. The current therapeutic strategy for glioma mainly consists of surgical resection, radiotherapy, chemotherapy and the use of anti-angiogenesis agents. However, the prognosis of patients is poor, and glioma is associated with high mortality. Potential underlying molecular mechanisms and the potential biomarkers of gliomas have been explored by identifying key genes and pathways, such as isocitric dehydrogenase (IDH) 1/2, 1p/19q co-del, TP53, and PTEN [3, 4]. However, the underlying molecular mechanism for glioma tumorigenesis has not been fully established, as it is associated with a number of contributing oncogenes. Therefore, effective biomarkers are required to improve the prognosis of patients with gliomas.

Pantothenate kinase 1 (PANK1) is implicated in hepatic coenzyme A (CoA) synthesis during the transition from glucose utilization to fatty acid oxidation that occurs in the fasting state [5]. A phosphopantothenate replacement therapy to bypass the genetic deficiency in the Pank1−/− mouse model recently showed that administration of selected PANK1-targeted compounds alleviated this deficiency in hepatic CoA [6]. PANK1‐deficient mice were unable to convert pyruvate, oxaloacetate or glycerol into glucose. These findings showed that glucose metabolism is significantly associated with the prognosis of patients with glioblastoma [7]. PANK1 plays an important role in mediating CoA biosynthesis; therefore, PANK1 expression may be correlated with the occurrence, development, and prognosis of glioblastoma. PANK1 mRNA is enriched in several metabolic processes, implying that it plays a potential role in metabolic programming in cancers [8, 9]. Currently, only a few studies have explored the role of PANK1 in cancer [10]. To the best of our knowledge, this is the first study to explore the expression and prognostic role of PANK1 in gliomas.

## 2 MATERIALS AND METHODS

### 2.1 Glioma Samples

A total of 56 human glioma tissues and 10 non-tumor brain tissues were collected during surgeries of glioma patients. Patients included in this study were admitted to Renmin Hospital of Wuhan University between September 2015 and December 2020. The patients included 2 cases of World Health Organization (WHO) level I glioma, 16 cases of grade II glioma, 12 cases of level III glioma, and 26 cases of GBM. All patients had primary lesions, and none of them underwent chemotherapy or radiotherapy before the operation. All patients signed an informed consent agreement form. Ethical approval was obtained from the Renmin Hospital of Wuhan University.

### 2.2 Bioinformatics Analysis

Data were retrieved from The Cancer Genome Atlas (TCGA) through the Gliovis tool (http://gliovis.bioinfo.cnio.es/) [11]. Rembrandt and Gravendeel (GSE16011) datasets were retrieved using the Gliovis webserver. In addition, glioma data were retrieved from the Chinese Glioma Genome Atlas (CGGA, http://www.cgga.org.cn/index.jsp), which consists of 693 glioma tissues [12]. Oncomine (https://www.oncomine.org) is an online cancer database that comprises gene expression array data [13]. PANK1 co-expressed genes were analyzed using cBioPortal (http://www.cbioportal.org/) [14]. Correlated genes with Spearman | r |≥0.5 and *P <* 0.01 were considered significant. Gene ontology (GO) analysis was performed to explore the most-related genes using the Metascape tool (http://metascape.org).

### 2.3 RNA Isolation and RT-PCR

Total RNA was extracted using TRIzol reagent (Invitrogen, USA) from glioma cells. cDNA was prepared using the PrimeScript RT reagent kit (Takara), and SYBR Green II Mixture (TaKaRa, Japan) was used for real-time PCR. All procedures were performed following the manufacturer’s protocol. The specific primer pairs used in this study were as follows: PANK1_F: 5’-ACGTCGAACCGGACTCTG-3’, R: 5’-CGTCTTGCGATCTCTCAGCT-3’; MMP2_F: 5’-ATCA GTTACCACGACGCATC-3’, R: 5’-GCTCTACGCAA CTGTCTGGT-3’; Vimentin_F: 5’-ATCTGACAATTCC AGCTCGAA-3’, R: 5’-ATTCGGATCGACGTCATGGCT-3’; GAPDH_F: 5’-GGCTAGTCCATCACGGTTATGA-3’; R:5’-GCTATACCAGATGGAAGAC-3’.

### 2.4 Immunohistochemistry (IHC) Staining and Analysis

Tissues underwent deparaffinization and were rehydrated to retrieve antigens, and endogenous peroxidase activity was inhibited, then tissue sections were blocked using 4% paraformaldehyde. Tissue sections were then incubated with primary antibodies (anti-PANK1, 1:500, Santa Cruz, sc-390865; VIM, 1:200, Proteintech, 10366-1-AP) overnight. After washing with phosphate-buffered solution (PBS), tissue sections were incubated in biotinylated polyclonal secondary antibody (reagent C) at room temperature for 30 min, followed by washing with PBS. Diaminobenzidine (DAB) substrate was then added for development, followed by hematoxylin counter-staining, dehydration, resin mounting and microscopic observation. Two observers scored the IHC staining independently, and a third observer decided in case of discrepancy. Positive staining (%) was as follows: score 0, no staining; score 1, 1%-25%; score 2, 26%-50%; score 3, 51-75%; score 4, >75%). The staining intensity scores were: 0, negative staining; 1, weakly positive staining; 2, moderately positive staining; 3, highly positive staining. Score definition = intensity score × percentage score. PANK1 protein expression was then classified as high (score 5-12) or low (score <4) expression.

### 2.5 Cell Culture and Transfection

Cells were cultured with Dulbecco's modified eagle medium (DMEM) and maintained in a humidified incubator with 5% CO_2_ at 37°C. The cells in the logarithmic growth phase were seeded and cultured in six‐well plates. The cells were transfected with a mixture of Lipo3000 containing 2 μg of each plasmid (Pank1 overexpression and PcDNA 3.1) in antibiotic-free media. Transfected cells were then collected and cultured for further use. All experiments were performed in triplicates.

### 2.6 Transwell Assay

Overexpression plasmid targeting PANK1 and negative control transfected cells were seeded on a collagen-based matrix, and the collagen-based matrix was covered. Serum-free medium was added to the upper chambers, whereas serum medium was added to the lower chambers. After culturing for 48 hours in an incubator with 5% CO_2_ at 37°C, the cells on the upper surface of the transwell were collected with a cotton swab. Cells were fixed with 4% paraformaldehyde for 15 min. Slides were washed three times with PBS, and then cells were stained with 0.1% crystal violet for 10 min. Stained cells from five different fields were photographed under an inverted microscope, and the cell numbers were calculated.

### 2.7 Cell Counting Kit-8 Assay

Cell viability was monitored using Cell Counting Kit-8 (CCK8) (CCK8, Dojindo, Japan) following the manufacturer’s instructions. Transfected glioma cells (3000 cells/well) were seeded into 96-well plates. Three parallel holes were set for each group. After incubation at 37°C for 24, 48, and 72 h, 10 μl of CCK8 reagent was added to each well. CCK8 assay (Dojindo) was performed, and the optical density (OD) at 450 nM was determined using an automatic microplate reader (BioTek).

### 2.8 Statistical Analysis

Continuous variables were represented as Mean ± standard deviation (SD). Comparisons between the glioma and normal tissues were performed using independent sample t-tests, and One-Way ANOVA analysis was used for three or more group comparisons. The relationship between PANK1 expression and survival time in glioma patients was assessed by Kaplan-Meier analysis. Univariate and multivariate Cox analyses were performed to explore prognostic-related independent risk factors. Statistical difference was defined as *P<*0.05. All statistical analyses were performed using SPSS 21, and graphics were generated using GraphPad 8.0.

## 3 RESULTS

### 3.1 PANK1 Expression Decreased in Glioma Tissues

**Fig. (1):**
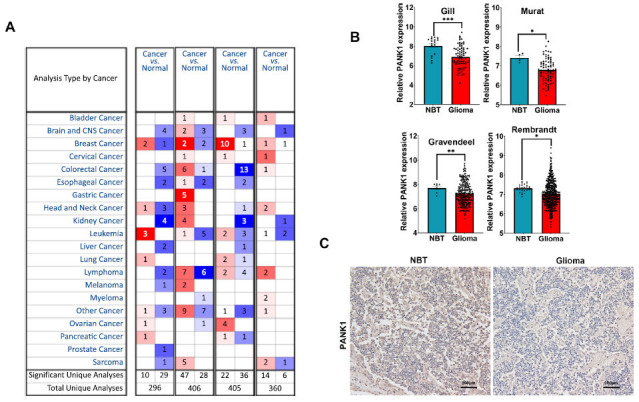
PANK1 expression decreased in glioma tissues. (**A** and **B**) Expression of PANK family in glioma using Oncomine, Gill, Murat, Gravendeel and Rembrandt datasets; (**C**) Immunohistochemistry (IHC) staining analysis of PANK1 expression in glioma tissues and non-tumor brain tissues (NBT).*, *P<*0.05, **, *P<*0.01, ***, *P<*0.001.

Oncomine and four other public datasets, namely Gill, Murat, Gravendeel, and Rembrandt, were used to explore PANK gene expressions in glioma patients. Analysis showed that PANK1 expression was significantly downregulated in malignant brain tumors compared with normal brain tissues (Figs. **1A** and **B**). IHC was performed to further explore the role of PANK1 in glioma. IHC analysis showed that the level of PANK1 was significantly lower in 56 glioma tissues compared with the level in non-tumor brain tissues (Fig. **1C**).

### 3.2 PANK1 Expression is Negatively Associated with Glioma WHO Grade

**Fig. (2):**
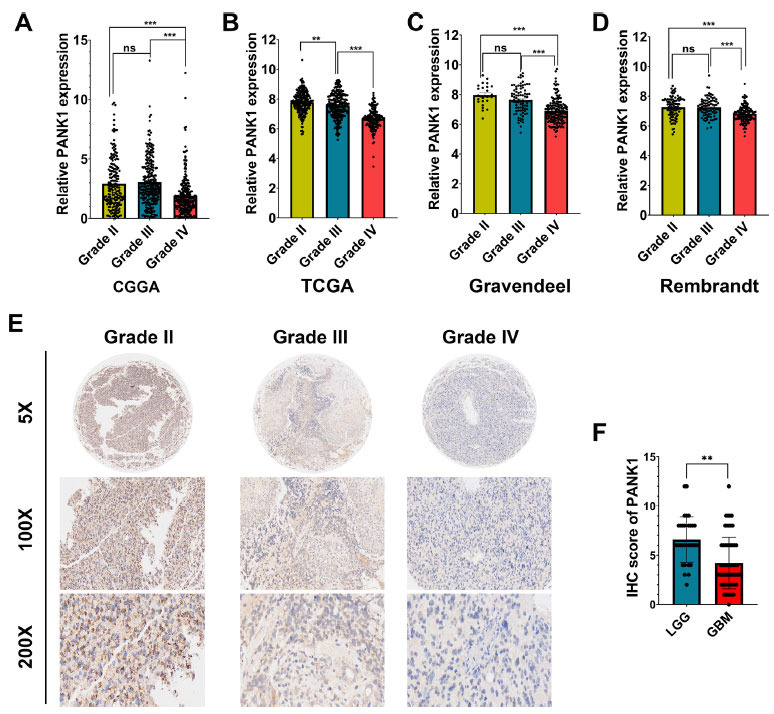
PANK1 expression is negatively associated with glioma WHO grade. (**A-D**) normalized mRNA expression of PANK1 in different grades of glioma based on TCGA, CGGA, Gravendeel, and Rembrandt datasets; (**E** and **F**) IHC was performed to determine protein level of PANK1 in the in-house cohort. LGG, lower grade glioma; GBM, glioblastoma; **, *P<*0.01, ***, *P<*0.001; ns, no significance.

Gliomas can be categorized into low-grade and high-grade (also called malignant tumor) tumors based on their malignancy degree. PANK1 expression significantly decreased with an increase in tumor grade in CGGA, TCGA, Gravendeel and Rembrandt datasets (Fig. **2A-D**). IHC staining was performed to further explore the relationship between PANK1 expression and glioma grade. IHC analysis of 38 glioblastomas (GBM, WHO IV) tissues and 18 lower-grade glioma tissues (LGG, WHO I-III) showed that the PANK1 expression level was lower in GBM compared with that in LGG (Figs. **2E** and **F**).

### 3.3 PANK1 Expression Associated with Glioma Molecular **Characteristic**

**Fig. (3):**
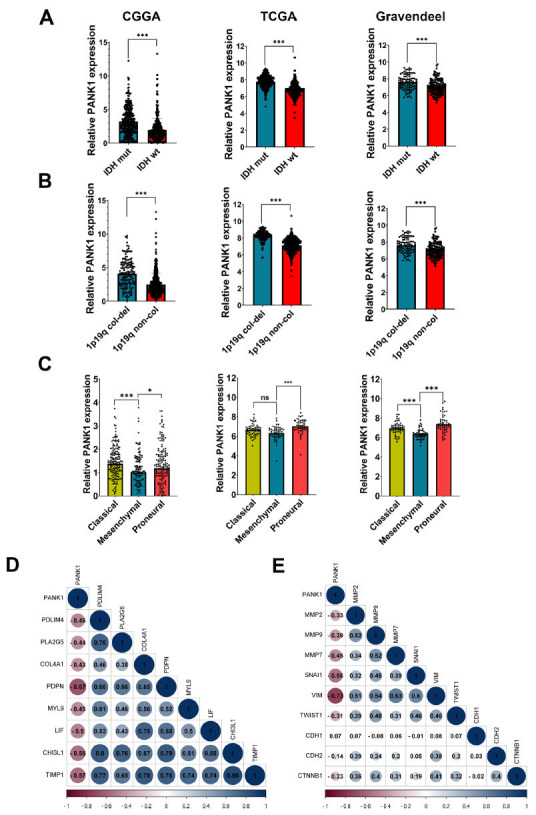
PANK1 expression associated with glioma molecular characteristic. (**A-C**) Expression level of PANK1 in glioma patients with different IDH1/2,1p19q codeletion status and molecular subtypes using CGGA, TCGA and Gravendeel datasets; (**D** and **E**) Correlations between PANK1 and mesenchymal-related genes or invasion-related genes in TCGA, a ***, *P<*0.001, ns, no significance.

Patients with IDH-mutant gliomas showed better prognosis than those with wild-type IDH regardless of glioma grade or histological characteristics. Analysis of TCGA, CGGA and Gravendeel datasets showed significantly lower PANK1 mRNA expression level in the IDH-wildtype (WT) compared with the level in the IDH-mutant (Mut) gliomas in all grades (Fig. **3A**). Furthermore, the analysis showed that the 1p/19q non-codeletion was associated with lower PANK1 expression in TCGA, CGGA, and Gravendeel datasets (Fig. **3B**). Moreover, GBM has been grouped into four subtypes: classical, neural, proneural, and mesenchymal (ME). Mesenchymal subtypes are often associated with drug resistance and a worse prognosis. We found that PANK1 expression was decreased in the ME subtype compared with other subtypes (Fig. **3C**). Apparent correlations were observed between the mRNA expression levels of PANK1 and ME-related genes, as well as invasion-related markers in the TCGA dataset (Figs. **3D** and **E**). These findings indicated that PANK1 is negatively correlated with glioma malignancy and may serve as a tumor suppressor gene.

### 3.4 Low PANK1 Expression Correlates with Worse Prognosis of Glioma Patients

**Fig. (4):**
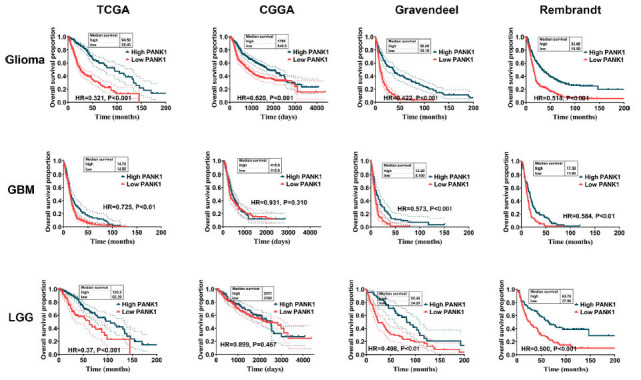
Low PANK1 expression level is correlated with worse prognosis of glioma patients. Relationship between PANK1 expression and prognosis of glioma patients based on TCGA, CGGA, Gravendeel and Rembrandt datasets. **Abbreviations:** LGG, lower grade glioma; GBM, glioblastoma. HR, hazard ratio.

The relationship between PANK1 expression and the patient prognosis was explored using Kaplan–Meier analysis. Analysis showed that patients with high expression levels of PANK1 had a significantly better prognosis compared with those with low expression levels of PANK1 (Fig. **4**). Multivariate Cox regression analysis showed that low expression of PANK1 was an independent factor associated with short overall survival in glioma patients in TCGA dataset (Table **1**). Next, we divided glioma into two groups, namely lower grade glioma (LGG) and GBM. We found that both LGG and GBM patients with high expression of PANK1 had a significantly better prognosis than those with low expression of PANK1 in TCGA, Gravendeel and Rembrandt datasets (all *P <*0.01). While no significance was observed in CGGA (*P>*0.05).

### 3.5 GO Enrichment Analysis and Pathway Prediction of PANK1

**Fig. (5):**
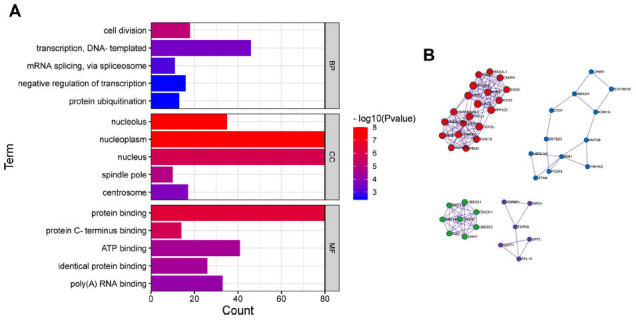
GO enrichment analysis and pathway prediction of PANK1 in glioma. PANK1 co-expressed genes were analyzed using cBioPortal (http://www.cbioportal.org/).Correlated Genes with Spearman | r |≥0.5 and *P <* 0.01 were considered to have significance. GO functional enrichment analysis (**A**) and protein-protein interaction analysis (**B**) were performed using Metascape (http://metascape.org). **Abbreviations:** BP, biological process; MF, molecular function; CC, cellular component.

PANK1 co-expressed genes were analyzed using cBioPortal (http://www.cbioportal.org/). Through the cBioPortal database, 5251 genes significantly coexpressing with PANK1 in TCGA-GBM samples were obtained. Analysis showed that the top three biological processes were involved in cell division, transcription, DNA-templated and mRNA splicing *via* spliceosome (Fig. **5A**). Enriched GO terms for molecular function category were protein binding, protein C-terminus binding, and ATP binding. Enriched GO terms for the cellular components category were related to the nucleolus, nucleoplasm, and nucleus (Fig. **5A**). Networks showing the predicted protein-protein interactions of the related genes enriched pathways of the top 4 modules are shown in Fig. (**5B**).

### 3.6 PANK1 is Associated with Immune Cell Infiltration in Glioma

**Fig. (6):**
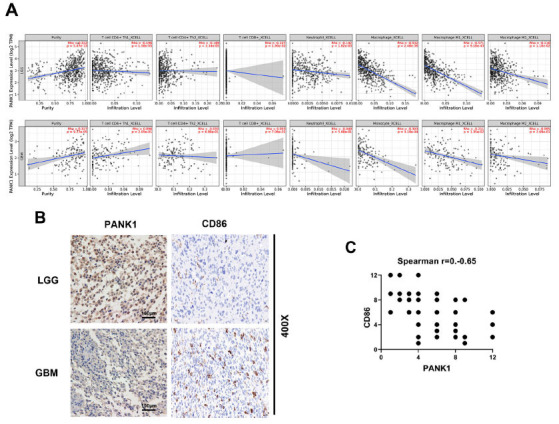
PANK1 is associated with immune cell infiltration in glioma. (**A**) Spearman correlation analysis between PANK1 expression and the immune infiltration levels from Tumor Immune Estimation Resource (TIMER). (**B-C**) Representative images of IHC staining of PANK1 and CD86 in our cohort. **Abbreviations:** LGG, lower grade glioma; GBM, glioblastoma.

Immune cell population infiltration has an impact on glioma progression. We evaluated the correlation between PANK1 expression and the immune infiltration levels from Tumor Immune Estimation Resource (TIMER, https://cistrome.shinyapps.io/timer/). As the results shown in Fig. (**6A**), we found that the expression levels of PANK1 significantly correlated with the infiltration of macrophage both in LGG and GBM. The expression of PANK1 in the high immune cell infiltration group was significantly lower than that in the low immune cell infiltration group using the M1 macrophage marker, CD86, in glioma tissues (Spearman r= -0.65, *P<*0.01, Figs. **6B** and **C**).

### 3.7 PANK1 Inhibits the Proliferation and Invasion of Glioma Cells *in vitro*

**Fig. (7):**
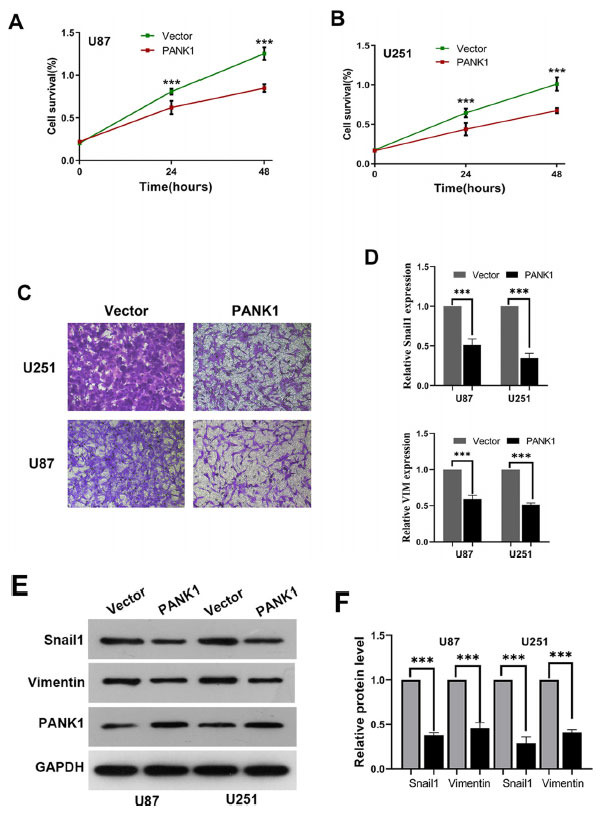
PANK1 inhibited the proliferation and invasion of glioma cells *in vitro.* (**A-B**) Cell proliferation of U87 and U251 cells using CCK8 assays. (**C**) Invasive capacity of glioma cells transfected with overexpression PANK1 plasmid using transwell assay. (**D-F**) Real-time quantitative PCR and western blot analysis showing induction of Snail1 and Vimentin expression by PANK1 overexpression. ***, *P<*0.001.

Cell proliferation was determined using CCK8 assays. Analysis showed that overexpression of PANK1 inhibited cell proliferation in U87 and U251 cells (Figs. **7A** and **B**). Transwell assay showed that overexpression of PANK1 significantly decreased the invasive capacity of glioma cells (Figs. **7C** and **D**). Moreover, Real-time quantitative PCR analysis and Western blot showed that PANK1 overexpression induced snail1 and Vimentin expression (Figs. **7E** and **F**).

## 4 DISCUSSION

Only a few studies have explored the role of PANK1 in cancer. To the best of our knowledge, the current study is the first to report that expression of PANK1 is downregulated in glioma samples and negatively correlated with tumor malignancy. The decreased expression levels of PANK1 were found to be significantly correlated with poor prognosis of glioma, implying that it is a potential biochemical marker in glioma. PANK1 is implicated in regulating the extracellular matrix and analysis showed that PANK1 may be a crucial gene involved in regulating the proliferation and invasion of glioma cells.

A previous study explored PANK family expression in acute myeloid leukemia and reported that PANK1 expression was not significantly correlated with event-free survival (EFS) and overall survival (OS) of patients [15]. Recent studies have reported that the metabolic characteristics of gliomas may be related to their tumor origins, and remodeling energy metabolism is a promising strategy for the management of gliomas [16-18]. PANK1 is associated with coenzyme A (CoA) synthesis and metabolism-related diseases [5]. CoA activates intermediates of the tricarboxylic acid (TCA) cycle, which is implicated in glioma malignancy progression [19]. Therefore, it is important to explore the molecular mechanisms underlying PANK1 expression in the glioma malignancy process to help identify novel therapeutic targets for glioma. The findings of this study showed that PANK1 expression was significantly downregulated in glioma tissues compared to normal brain tissues. In addition, the PANK1 expression level decreased with an increase in glioma grade and tumor malignancy progression. IDH plays an important role in cell metabolism and is a key component of the tricarboxylic acid (TCA) cycle. Previous studies reported that the IDH mutation affects metabolic processes in glioma cells, and the wildtype promotes tumor cells and accelerates growth [20, 21]. In this study, PANK1 was significantly differentially expressed in IDH mutant and IDH wildtype glioma tissues. These findings imply that PANK1 is a novel tumor suppressor gene and might play an important role in the regulation of tumor metabolism.

The findings of the present study provide a new perspective on the functions of PANK1 in glioma cells. Functional enrichment analyses indicated that PANK1 mainly participates in extracellular structure organization. Glioma invasion occurs through the secretion of MMPs into the tumor microenvironment and the degradation of extracellular matrix [22]. These results indicated that PANK1 might be involved in regulating the invasion of glioma cells. Correlation analysis showed a significant correlation between PANK1 and invasion-related markers, such as vimentin, snail1 and mmp2. PANK1 plays important roles in metabolism, and metabolites produced by tumor cells significantly affect tumor migration and invasion. PANK1 may promote glioma cell invasion by regulating tumor cell metabolism. However, further studies should explore the mechanism of action of PANK1 in promoting glioma cell invasion.

In summary, PANK1 expression is downregulated in glioma tissues and is negatively correlated with tumor malignancy. High expression of PANK1 is correlated with significantly better prognosis of glioma patients compared to glioma patients with low expression levels of PANK1. Moreover, PANK1 may be a key regulator of the proliferation and invasion of glioma cells. These findings showed that PANK1 is a novel biomarker and a potential target for the treatment of glioma.

## CONCLUSION

PANK1 expression was downregulated in glioma tissues and negatively correlated with glioma malignancy. Lower PANK1 expression is associated with a worse prognosis of glioma patients, indicating that PANK1 is a novel prognostic biomarker in glioma patients.

## Figures and Tables

**Table 1:** Univariate and multivariate cox regression analysis of of prognostic parameters in TCGA.

	**Univariate Cox Regression**		**Multivariate Cox Regression**	
	**HR (95%CI)**	***P* value**	**HR (95%CI)**	***P* value**
Age (≥55y *vs.* <55y)	0.62 (0.51-0.82)	<0.01	0.72(0.61-0.98)	0.47
Gender (Female *vs.* male)	1.32 (0.92-1.57)	0.74	-	-
Grade (IV *vs*. I-III)	2.34 (1.72-5.62)	<0.01	1.89(1.20-3.17)	<0.01
IDH1 status (wildtype *vs*. mutation)	4.10 (0.07-0.13)	<0.01	2.16(1.14-3.41)	<0.01
MGMT promoter	1.51 (1.22-3.68)	0.13	-	-
Subtype (ME *vs*. others)	3.08 (1.95-6.52)	<0.01	2.32(1.41-2.80)	0.01
PANK1 expression (Low *vs.* High)	1.20 (1.10-2.04)	<0.01	1.34 (1.21-1.48)	<0.01

## Data Availability

The authors that the data and supportive information are available within the article.

## LIST OF ABBREVIATIONS

TCA: Tricarboxylic Acid
CoA: Coenzyme A
EFS: Event-Free Survival
OS: Overall Survival

## References

[1] Ostrom Q.T., Patil N., Cioffi G., Waite K., Kruchko C., Barnholtz-Sloan J.S. (2020). CBTRUS statistical report: Primary brain and other central nervous system tumors diagnosed in the United States in 2013–2017.. Neuro-oncol..

[2] Brown T.J., Brennan M.C., Li M., Church E.W., Brandmeir N.J., Rakszawski K.L., Patel A.S., Rizk E.B., Suki D., Sawaya R., Glantz M. (2016). Association of the extent of resection with survival in glioblastoma.. JAMA Oncol..

[3] Eckel-Passow J.E., Lachance D.H., Molinaro A.M., Walsh K.M., Decker P.A., Sicotte H., Pekmezci M., Rice T., Kosel M.L., Smirnov I.V., Sarkar G., Caron A.A., Kollmeyer T.M., Praska C.E., Chada A.R., Halder C., Hansen H.M., McCoy L.S., Bracci P.M., Marshall R., Zheng S., Reis G.F., Pico A.R., O’Neill B.P., Buckner J.C., Giannini C., Huse J.T., Perry A., Tihan T., Berger M.S., Chang S.M., Prados M.D., Wiemels J., Wiencke J.K., Wrensch M.R., Jenkins R.B. (2015). Glioma groups based on 1p/19q, IDH, and TERT promoter mutations in tumors.. N. Engl. J. Med..

[4] Molinari E., Curran O.E., Grant R. (2019). Clinical importance of molecular markers of adult diffuse glioma.. Pract. Neurol..

[5] Leonardi R., Rehg J.E., Rock C.O., Jackowski S. (2010). Pantothenate kinase 1 is required to support the metabolic transition from the fed to the fasted state.. PLoS One.

[6] Leonardi R., Rock C.O., Jackowski S. (2014). Pank1 deletion in leptin-deficient mice reduces hyperglycaemia and hyperinsulinaemia and modifies global metabolism without affecting insulin resistance.. Diabetologia.

[7] Caniglia J.L., Jalasutram A., Asuthkar S., Sahagun J., Park S., Ravindra A., Tsung A.J., Guda M.R., Velpula K.K. (2021). Beyond glucose: Alternative sources of energy in glioblastoma.. Theranostics.

[8] Yang L., Zhang B., Wang X., Liu Z., Li J., Zhang S., Gu X., Jia M., Guo H., Feng N., Fan R., Xie M., Pei J., Chen L. (2020). P53/PANK1/miR‐107 signalling pathway spans the gap between metabolic reprogramming and insulin resistance induced by high‐fat diet.. J. Cell. Mol. Med..

[9] Wang S.J., Yu G., Jiang L., Li T., Lin Q., Tang Y., Gu W. (2013). p53-dependent regulation of metabolic function through transcriptional activation of pantothenate kinase-1 gene.. Cell Cycle.

[10] Liu Y., Cheng Z., Li Q., Pang Y., Cui L., Qian T., Quan L., Dai Y., Jiao Y., Zhang Z., Ye X., Shi J., Fu L. (2019). Prognostic significance of the PANK family expression in acute myeloid leukemia.. Ann. Transl. Med..

[11] Bowman R.L., Wang Q., Carro A., Verhaak R.G.W., Squatrito M. (2017). GlioVis data portal for visualization and analysis of brain tumor expression datasets.. Neuro-oncol..

[12] Zhao Z., Zhang K.N., Wang Q., Li G., Zeng F., Zhang Y., Wu F., Chai R., Wang Z., Zhang C., Zhang W., Bao Z., Jiang T. (2021). Chinese glioma genome atlas (CGGA): A comprehensive resource with functional genomic data from chinese glioma patients.. Genomics Proteomics Bioinformatics.

[13] Rhodes D.R., Yu J., Shanker K., Deshpande N., Varambally R., Ghosh D., Barrette T., Pander A., Chinnaiyan A.M. (2004). ONCOMINE: A cancer microarray database and integrated data-mining platform.. Neoplasia.

[14] Cerami E., Gao J., Dogrusoz U., Gross B.E., Sumer S.O., Aksoy B.A., Jacobsen A., Byrne C.J., Heuer M.L., Larsson E., Antipin Y., Reva B., Goldberg A.P., Sander C., Schultz N. (2012). The cBio cancer genomics portal: An open platform for exploring multidimensional cancer genomics data.. Cancer Discov..

[15] Valk P.J.M., Verhaak R.G.W., Beijen M.A., Erpelinck C.A.J., van Doorn-Khosrovani S.B.W., Boer J.M., Beverloo H.B., Moorhouse M.J., van der Spek P.J., Löwenberg B., Delwel R. (2004). Prognostically useful gene-expression profiles in acute myeloid leukemia.. N. Engl. J. Med..

[16] Michelakis E.D., Sutendra G., Dromparis P., Webster L., Haromy A., Niven E., Maguire C., Gammer T.L., Mackey J.R., Fulton D., Abdulkarim B., McMurtry M.S., Petruk K.C. (2010). Metabolic modulation of glioblastoma with dichloroacetate.. Sci. Transl. Med..

[17] Garcia J.H., Jain S., Aghi M.K. (2021). Metabolic drivers of invasion in glioblastoma.. Front. Cell Dev. Biol..

[18] Kesarwani P., Prabhu A., Kant S., Chinnaiyan P. (2019). Metabolic remodeling contributes towards an immune-suppressive phenotype in glioblastoma.. Cancer Immunol. Immunother..

[19] Rock C.O., Calder R.B., Karim M.A., Jackowski S. (2000). Pantothenate kinase regulation of the intracellular concentration of coenzyme A.. J. Biol. Chem..

[20] Touat M., Idbaih A., Sanson M., Ligon K.L. (2017). Glioblastoma targeted therapy: Updated approaches from recent biological insights.. Ann. Oncol..

[21] Pathmanapan S., Ilkayeva O., Martin J.T., Loe A.K.H., Zhang H., Zhang G.F., Newgard C.B., Wunder J.S., Alman B.A. (2021). Mutant IDH and non-mutant chondrosarcomas display distinct cellular metabolomes.. Cancer Metab..

[22] Cuddapah V.A., Robel S., Watkins S., Sontheimer H. (2014). A neurocentric perspective on glioma invasion.. Nat. Rev. Neurosci..

